# Combination of *Bacillus* and Low Fertigation Input Promoted the Growth and Productivity of Chinese Cabbage and Enriched Beneficial Rhizosphere Bacteria *Lechevalieria*

**DOI:** 10.3390/biology12081130

**Published:** 2023-08-14

**Authors:** Shi-Chang Zhang, Yu-Lu Zhang, Xiao-Jing Guo, Ming Luo, Shi-Dong Li, Rong-Jun Guo

**Affiliations:** 1 State Key Laboratory for Biology of Plant Diseases and Insect Pests, Institute of Plant Protection, Chinese Academy of Agricultural Sciences, Beijing 100193, China; 2 College of Agriculture, Xinjiang Agricultural University, Urumqi 830052, China

**Keywords:** soil water potential, growth promotion, *Bacillus*, rhizosphere microbial community, plant hormone

## Abstract

**Simple Summary:**

Chinese cabbage is often threatened by soft rot disease caused by *Pectobacterium* spp., which becomes severe due to overirrigation or heavy rainfall. An improved application technique of biocontrol *Bacillus* agents with low fertigation input is suggested as an environment-friendly strategy to control soft rot disease and improve the cabbage yield. In this study, the effect of *Bacillus velezensis* B006 on cabbage growth under normal and low fertigation conditions was firstly evaluated, and then the relationships of the plant growth with the cabbage rhizosphere microbial communities and the production of endogenous hormones in cabbage roots were studied. Our results highlighted that B006 application combined with low fertigation input promoted cabbage growth and increased root vitality which were positively related to enrichment of *Lechevalieria* in the cabbage rhizosphere and the production of indole-3-acetic acid and salicylic acid in cabbage roots. In conclusion, we provide supporting evidence on the improvement of *Bacillus* application in fields to promote the healthy growth and productivity of cabbage.

**Abstract:**

Long-term overfertilization increases soil salinity and disease occurrence and reduces crop yield. Integrated application of microbial agents with low fertigation input might be a sustainable and cost-effective strategy. Herein, the promoting effects of *Bacillus velezensis* B006 on the growth of Chinese cabbage under different fertigation conditions in field trials were studied and the underlying mechanisms were revealed. In comparison with normal fertigation (water potential of −30 kPa and soluble N, P, K of 29.75, 8.26, 21.48 Kg hm^−2^) without B006 application, the combination of *B. velezensis* B006 and reduced fertigation input (−50 kPa and N, P, K of 11.75, 3.26, 6.48 Kg hm^−2^) promoted cabbage growth and root development, restrained the occurrence of soft rot disease, and improved the yield. High-performance liquid chromatography (HPLC) analyses indicated that B006 application promoted the production of indole-3-acetic acid and salicylic acid in cabbage roots, which are closely related to plant growth. Rhizosphere microbiota analyses indicated that the combination of low fertigation input and B006 application promoted the enrichment of *Streptomyces*, *Lechevalieria*, *Promicromonospora*, and *Aeromicrobium* and the abundance of *Lechevalieria* was positively correlated with the root length and vitality. This suggested that the integrated application of reduced fertigation and *Bacillus* is highly efficient to improve soil ecology and productivity and will benefit the sustainable development of crop cultivation in a cost-effective way.

## 1. Introduction

Water and fertilizer inputs in the soil at certain amounts are essential for normal plant growth and development. However, their deficiencies or overinputs disrupt soil ecology, affect plant physiology, increase the occurrence of diseases, and correspondingly reduce the yield [1,2,3,4]. In comparison to the traditional water and fertilizer managing method [5], fertigation is a more promising solution to combine irrigation and fertilization to save water and fertilizer and facilitate plant development. It provides appropriate and cost-effective inputs of water and fertilizer with better targeted timing and controls soil moisture and nutrients as well as soil degradation and saline–alkali risks [6,7,8]. In a tomato fertigation system, the reduction of water and fertilizer inputs by 25.3% and 40.6%, respectively, resulted in a yield increase of 9.0% and a profit increase of 9.3% [9]. In Israel, the utilization efficiency of water and fertilizer could be improved by 40–60% and 30–50%, respectively, by using the fertigation approach [10]. 

Plant roots have strong plasticity to adapt to the soil and environmental changes [11]. In response to water stress, plant can elongate roots into deep soil to enlarge the root–soil interface, increase the root/shoot ratio, enhance root vitality, improve root development, and promote the absorption of water and nutrients from soil [12,13,14]. Moreover, middle-level drought can enhance plant tolerance through reducing water loss, promoting root sheath formation, and increasing root length [15]. Therefore, root length has been suggested to be an indicator of plant tolerance to drought. In addition to water stress, low inputs of nitrogen and phosphorus can induce the growth of primary roots and enhance the lateral and vertical development of roots as well as the formation of root hair [16,17]. Additionally, the water content and nutrients of soil are also closely related to the occurrence of bacterial diseases. For example, high soil moisture increased the incidence of tomato and ginger wilt diseases caused by *Ralstonia solanacearum* [1,18], while lower water and fertilizer inputs promoted plant growth and the production of some antipathogenic chemicals in rice [19]. However, long-term water stress may lead to yield reduction [20]. Therefore, it is of great importance to balance the water and fertilizer inputs with the alleviation of plant diseases and improved yield. 

In agroecosystems, soil microbial communities are closely associated with plant growth, nutrient cycling, soil structure, and so on. It is well known that the occurrence of plant disease is affected by environmental factors through direct effects on the pathogen or indirect effects via affecting plant immunity and defense hormone pathways [21]. Of them, rhizosphere microbiota forms the first line to defend against pathogens and reduce disease severity [22] through intense competition for nutrients, space, and other resources [23,24] or through secreting antimicrobial molecules [25]. Soil water not only influences the plant root development and distribution, but also affects the interactions among plants–soil–microbes and shapes the microbial community correspondingly. It was reported that some strains of Firmicutes and Actinomycetes could survive in drought-stressed soil [26,27,28] and the enrichment of *Streptomyces* could improve plant tolerance to drought stress, promote crop growth, and increase the yield [29]. Besides soil water, fertilization strategies also have crucial effects on soil microbiota and plant health. For example, organic fertilizer affected the compositions of bacterial and fungal communities in the root-associated soil and rhizosphere, such as the enrichment of plant-beneficial *Talaromyces* in the rhizosphere, and increased the expression levels of defense-related genes in shoots of lettuce [30]. 

Chinese cabbage (*Brassica rapa* L. ssp. *pekinensis*) is one of the most economically important vegetable in Asia, particularly in China, Korea, and Japan. During the whole growing period, it is often threatened by soft rot disease caused by *Pectobacterium* spp. [31], and the yield loss reaches up to 50% in seriously infested fields [32,33]. Overirrigation or heavy rainfall further promotes the spread and outbreak of soft rot disease [34,35]. Besides soil moisture, long-term nitrogen input is another factor closely related to the occurrence of bacterial wilt disease [1,23,36]. Thus, water and fertilizer management is suggested to be a promising strategy to control cabbage soft rot disease. When combined with biocontrol agents like *Bacillus*, the growth of eggplant and cabbage can be promoted even in a low fertigation input system [37,38]. *Bacillus velezensis* B006 is a biocontrol strain harboring a few lipopeptide-encoding genes and producing surfactin and fengycin with antagonistic activity against bacterial and fungal pathogens [39,40]. In this study, the effects of integrated application of fertigation and plant-beneficial *Bacillus* B006 on the growth and soft rot disease control of Chinese cabbage were tested in field trials and its effects on the rhizosphere microbiota and endogenous hormones were determined. The results will benefit the understanding of the interactions among fertigation–beneficial bacterium–plant and the underlying mechanism from viewpoints of soil ecology and plant physiology.

## 2. Materials and Methods

### 2.1. Materials

*B. velezensis* B006, conserved in our laboratory, was grown in M3 medium containing 17.5 g L^−1^ corn starch, 5.25 g L^−1^ sugar, 14.0 g L^−1^ soybean cake powder, and 5.25 g L^−1^ CaCO_3_ as described by Wang et al. [41]. *Pectobacterium carotovorum* subsp. *brasiliensis* BC1, the cabbage root rot causative agent, was provided by the Biotechnology Center of Beijing Academy of Agriculture and Forestry Sciences. 

Both organic fertilizer and water-soluble fertilizer were provided by Qigao Biology Science and Technology Co., Ltd. (Beijing, China). The organic matter in the organic fertilizer was over ≥45%, while the total amount of N/P/K in the water-soluble fertilizer was 36% at a ratio of 18/5/13. 

The Chinese cabbage variety Zhongshukuai No.1 was provided by the Zhongshu Seed Industry Technology Co., Ltd. (Beijing, China). 

### 2.2. Experimental Design 

A field experiment involving two factors (low vs. normal fertigation), each at two levels (pre-inoculated with B006 vs. not inoculated), was carried out in the greenhouse located at Changning Farm, Beijing. Corn was previously grown in the field, which contains a yellow loam soil. The treatment combinations were labeled LFW, LFWB, NFW, and NFWB, representing low fertigation without and with B006 application and normal fertigation without and with B006 application, respectively. The four treatment combinations were randomly allocated to four plots within each of four replications. Each replicate had four ridges with a width of 30 cm, and each ridge had one dripping band. The area of each field trial was 2.25 m × 4.0 m, and the areas of 2.5 m × 4.0 m at each side of the field served as protective lines. The furrow width between the ridges was 20 cm, the distance of dripping holes was 20 cm, and the tensiometer was set between two plants with a depth of 25 cm. The water potentials were set at −50 and −30 kPa in the low-fertigation (LFW) and normal-fertigation (NFW) treatments, respectively. The organic fertilizer was first applied to the soil at a dosage of 17.3 ton hm^−2^ before planting. Three to four cabbage seeds were sown in each planting hole containing 5 g of potting soil pre-inoculated with B006 (B) at a final concentration of 1.0 × 10^7^ CFU g^−1^ or not. After germination, seedlings were reduced to one in each planting hole. As shown in Table S1, the cabbages were irrigated with 199.1 m^3^ hm^−2^ of water and grew for 15 days till the water potential reached −30 kPa, and this period was defined as stage T1. At stage T2, the low-input treatment combinations (labeled LFW2 when no B006 was applied and LFWB2 when B006 was applied) were fertigated with reduced water potential and soluble fertilizer (−50 kPa of water potential and 62.50 Kg hm^−2^ of soluble fertilizer) for 10 days, while normal-input treatment combinations (labeled NFW2 when no B006 was applied and NFWB2 when B006 was applied) were fertigated with normal water potential and soluble fertilizer (−30 kPa of water potential and 165.25 Kg hm^−2^ of soluble fertilizer). Then, the cabbages were drenched with 50 mL of pathogen suspension (1 × 10^8^ CFU mL^−1^), and the water potential was maintained at −45 kPa for treatment combinations LFW3 and LFWB3 and −25 kPa for treatment combinations NFW3 and NFWB3 for 25 days (stage T3). 

### 2.3. Growth Performance and Root Vitality of Chinese Cabbage 

Eight cabbage seedlings were collected from each replicate of each treatment combination on the last day of stage T1 and used for the observation of plant growth, while six seedlings from each replicate of each treatment combination on the last day of stage T2 were collected for the assessment of growth performance. After measuring the heights and fresh weights of cabbage seedlings, the above-ground parts were dried at 105 °C for 1.5 h for the measurement of dry weights. The root lengths were measured using a ruler. Three root tips were mixed and ground into powder with 0.1 g of quartz sand in a sterile mortar and subjected to the determination of root vitality by using the triphenyl tetrazolium chloride (TTC) method [42]. 

### 2.4. Occurrence of Soft Rot Disease 

Twenty-five days after the inoculation of soft rot causative agent *P. carotovorum* subsp. *brasiliensis* BC1, the survival rate of cabbage seedlings, the severity of soft rot disease, and the cabbage yield were investigated. The disease level was classified as in Table 1. The relative control efficacy was calculated as (disease index of treatment combination NFW3 − disease index of other treatment combinations) × 100%/disease index in treatment combination NFW3. The average cabbage yield in each treatment combination was calculated based on the investigation of each trial (8.96 m^−2^). All data were statistically analyzed by SPSS 26.0. 

### 2.5. Determination of the Endogenous Hormones in Chinese Cabbage Roots 

#### 2.5.1. Standard Curves of ABA, IAA, SA, and ZT 

High-performance liquid chromatography (HPLC; Prominence LC-20A, Shimadzu, Japan) equipped with a GL-InertSustain-C18 reversed column (4.6 mm × 250 mm, 5 µm, GL Sciences, Japan) and a UV–Vis detector was used to analyze the endogenous hormones including abscisic acid (ABA), indole-3-acetic acid (IAA), salicylic acid (SA), and zeatin (ZT) in cabbage roots. Following the operation conditions set previously [43], water containing methanol (solution A) and 0.075% glacial acetic acid (solution B) at different ratios (7:3 for the first 20 min to 5:5 for the next 20–30 min) was used as the mobile phase with a flow rate of 1.0 mL min^−1^. The endogenous hormones were detected at 254 nm, the volume injected into the column was 10 µL, and the samples were analyzed at room temperature in triplicates. The retention times of ABA, IAA, SA, and ZT were 3.68, 12.25, 13.52, and 21.89 min, respectively. Different concentrations of ABA (0.05–1.0 µg mL^−1^), IAA (0.2–20.0 µg mL^−1^), SA (1.0–40.0 µg mL^−1^), and ZT (1.0–40.0 µg mL^−1^) were prepared and mixed, filtered through a 0.22 µm organic membrane, analyzed by HPLC, and used to construct the standard curves. 

#### 2.5.2. Analyses of the Endogenous Hormones in Cabbage Roots

The roots of 4 cabbage seedlings from each replicate of each treatment combination were collected at stage T1 and 3 seedlings were collected at stage T2 for the analyses of endogenous hormones. The endogenous hormones in the roots were extracted according to the method described by Zhao et al. [43]. Briefly, the fresh roots of each sample were cut and ground into powder with liquid nitrogen in a mortar. Five hundred milligrams of powdered roots in a 15 mL centrifuge tube was first extracted with 5 mL of ultrapure water:isopropanol:hydrochloric acid at a volume ratio of 1:2:0.002 ultrasonically (40 kHz at 4 °C for 30 min) and with agitation (100 r min^−1^ at 4 °C for 30 min). Another 8 mL of methylene dichloride was added, agitated at 100 r min^−1^ and at 4 °C for 30 min, and centrifuged at 4 °C, 5000 r min^−1^ for 30 min. Extracts in the lower layers were transferred into 20 mL brown bottles, blow-dried in N_2_, and dissolved in 1 mL of 80% methanol (containing 0.1% glacial acetic acid). The quality and quantity of each hormone in samples were determined based on the retention time and relative peak areas against the standard curves of ABA, IAA, SA, and ZT at concentrations ranging from 0.05~1.0 µg mL^−1^, 0.2~10.0 µg mL^−1^, 1.0~40.0 µg mL^−1^, and 1.0~40.0 µg mL^−1^, respectively, as described in Section 2.5.1. 

### 2.6. Analysis of the Rhizosphere Microbiota of Chinese Cabbage 

#### 2.6.1. Sample Preparation 

The soil samples were collected at different growing stages. Before sowing seeds, 2 g of bulk soil at a depth of 5–10 cm was collected from each ridge in a zigzag pattern [44], thoroughly mixed, and stored at −80 °C (S0). Using the same method, the cabbage seedlings were picked up and the rhizosphere soil tightly adhering to the roots of 3–4 cabbage seedlings was collected from each treatment before water stress (last day of stage T1) and after water stress (last day of stage T2) as described by Lundberg et al. [33]. A total of 36 samples were collected and used for the DNA extraction. 

#### 2.6.2. DNA Extraction and Illumina Miseq Sequencing 

Genomic DNA was extracted using the FastDNA^®^ SPIN Kit for Soil (MP Biomedicals, Santa Ana, CA, USA) and checked by electrophoresis in 1% agarose gel and spectrophotometry (A_260/280_). The DNA samples were stored at −20 °C before analysis. The V5–V7 hypervariable region of the bacterial 16S rRNA gene spanning approximately 394 bp was amplified with a two-step PCR amplification method. The first- and second-step amplification primers were 799F (5′-AACMGGATTAGATACCCKG-3′)/1392R (5′-ACGGGCGGTGTGTRC-3′) [45] and 799F/1193R (5′-ACGTCATCCCCACCTTCC-3′) [46,47], respectively. The first-step amplification conditions were as follows: initial denaturation at 95 °C for 3 min, followed by 27 cycles of denaturing at 95 °C for 30 s, annealing at 55 °C for 30 s and extension at 72 °C for 45 s, single extension at 72 °C for 10 min, and ending at 4 °C. The second-step amplification conditions were similar to those of the first step except for the 15 amplification cycles. The PCR mixtures contained 4 µL 5 × *TransStart* FastPfu (TranGen, Beijing, China) buffer, 2 µL 2.5 mM dNTPs, 0.8 µL each primer (5 µM), 0.4 µL *TransStart* FastPfu DNA Polymerase, 0.2 µL BSA, 10 ng template DNA, and ddH_2_O up to 20 µL. PCR reactions were performed in triplicate. The ITS1F (5′-CTTGGTCATTTAGAGGAAGTAA-3′)/ITS2R (5′-GCTGCGTTCTTCATCGATGC-3′) region of the fungal ITS gene spanning ~300 bp was amplified [48]. The PCR amplification system and conditions for the fungal ITS region were almost the same except for the 35 cycles in the first-step reaction. 

The PCR products were extracted from 2% agarose gel and purified using the AxyPrep DNA Gel Extraction Kit (Axygen Biosciences, Union City, CA, USA) according to the manufacturer’s instructions. After quantification by using QuantiFluor™-ST (Promega, Beijing, China), the PCR product of each sample was subjected to high-throughput sequencing on the Illumina Miseq platform according to the standard protocol provided by Majorbio Bio-Pharm Technology Co., Ltd. (Shanghai, China). Sterile RNase-free water was used as a negative control template in each PCR run. The raw reads were deposited into the NCBI Sequence Read Archive (SRA) database with the accession numbers of PRJNA879320 (bacterial microbiota) and PRJNA879910 (fungal microbiota). 

#### 2.6.3. Bioinformatic Analysis of the Soil Microbial Communities 

The 16S rRNA gene sequences were processed by using the Illumina Analysis Pipeline v4.0 developed by Majorbio. The demultiplexed raw DNA sequences were subjected to quality filtering with fastp (0.20.0) [49] based on sequence length and quality. Reads were truncated to a length of 300 bp, and only reads with a quality score of >20 and no ambiguous bases were retained for the analysis. The sequences that overlapped more than 10 bp were merged with FLASH (v1.2.11) [50], and read pairs which could not be assembled were discarded. After distinguishing the sequences of each sample according to the barcode and primers, the sequence direction was adjusted, the exact barcode was matched, and tag sequences and chimeric sequences were discarded. The sequences were then denoised using the DADA2 [51] plugin in the Qiime2 version 2020.2 [52] pipeline with recommended parameters (single nucleotide resolution based on error profiles) to give amplicon sequence variants (ASVs). To minimize the effects of sequencing depth on alpha and beta diversity measurements, the number of sequences from each sample was rarefied to 4841 ASVs. The taxonomy of each ASV was annotated by using the naive Bayes consensus taxonomy classifier against the SILVA 16S rRNA database (v138) with a confidential threshold of 70%. Those ASVs identified as mitochondria, chloroplasts, or cyanobacteria were removed.

The ITS gene sequences were processed using the same procedure as described above. The sequences from each sample were rarefied to 856 ASVs. Taxonomic assignment of ASVs was performed using the naive Bayes consensus taxonomy classifier and the unite8.0/its_fungi database.

The compositions of rhizosphere bacterial and fungal communities were compared among treatments. The bacterial phyla and genera with relative abundance over 1.0% and 1.5%, respectively, were identified. The fungal phyla and genera with relative abundance over 1.0% were identified. The abundance shifts of the top 15 genera were illustrated by a heatmap produced by Python 2.7 in R v3.3.1. Principal coordinate analysis (PCoA) [53] was performed to illustrate the variations of microbial communities based on the Bray–Curtis distance of ASVs using the vegan package, and permutational multivariate analysis of variance (PERMANOVA) [54] was used to determine the contribution of each factor to the rhizosphere microbial communities. The differential genera affected by water stress were determined by the comparison of their average relative abundance by using the Kruskal–Wallis H test and confirmed by the false discovery rate (FDR) (*p* ≤ 0.05). The top 20 bacterial genera of stage T2 were chosen for the Spearman correlation analysis with plant growth, fertigation input, and the endogenous hormones of stage T2 and illustrated by a heatmap (R 3.3.1, Python 2.7) [55].

## 3. Results

### 3.1. Growth Performance of Chinese Cabbage under Different Fertigation Conditions

**Figure 1:**
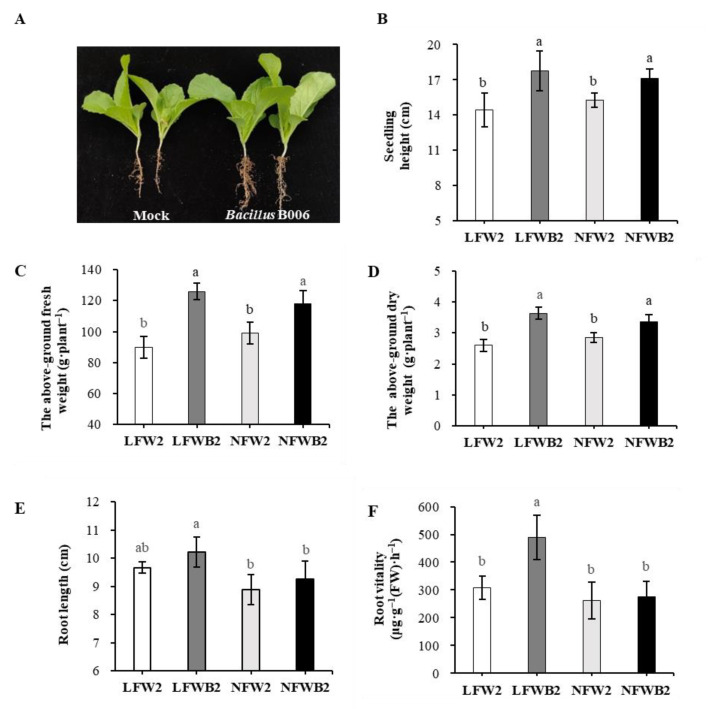
Growth performance of Chinese cabbage promoted by the integrated application of B006 and low fertigation input. (**A**) seedlings; (**B**) seedling height; (**C**) the above-ground fresh weight; (**D**) the above-ground dry weight; (**E**) root length; (**F**) root vitality. LFW, low fertigation input; NFW, normal fertigation input; B, application with B006; 2, collected at stage T2; Different letters above the columns indicate significant difference at 0.05 level.

The height, fresh and dry weights of the above-ground seedlings, root length, and root vitality showed differences across treatment combinations and development stages. As shown in Figure 1, the application of strain B006 to the rhizosphere promoted cabbage growth and root development at stage T1 (Figure 1A) and it significantly increased the seedling height (Figure 1B), fresh weight of the above-ground part of the seedlings (Figure 1C), and dry weight of the above-ground part of the seedlings (Figure 1D) by 12.3–40.0% at stage T2 (*p* < 0.05). Further comparison of the root length and root vitality indicated that low fertigation input combined with B006 application (treatment combination LFWB2) improved the root length compared to normal fertigation input treatment combinations (Figure 1E) and the root vitality compared to other treatment combinations (Figure 1F) significantly (*p* < 0.05) at stage T2. However, different fertigation levels had no significant effects on cabbage growth at stage T2 (*p* > 0.05) if strain B006 was not applied.

### 3.2. Combined B006 and Low Fertigation Input Suppressed Cabbage Soft Rot Disease

Two fertigation levels (low and normal) combined with B006 application were applied to control cabbage soft rot disease. As shown in Table 2, the occurrence of soft rot disease did not show significant difference between treatment combinations LFW and LFWB or between NFW and NFWB, but it was significantly lower under low-fertigation conditions (*p* < 0.05). When combined with the B006 application, the control efficacy reached up to 80.9%, 89.2% of the seedlings survived, and the highest yield of 4.3 × 10^4^ Kg hm^−2^ was achieved.

### 3.3. Endogenous Hormones in Cabbage Roots under Different Fertigation Conditions

The contents of four plant hormones, ABA, IAA, SA, and ZT, in cabbage roots were determined by using a reliable gradient HPLC-based method (Figure S1). Significant differences were detected between growth stages and across treatment combinations (Table 3). The SA contents were 9.83 to 18.43 µg g^−1^ at stage T1 and were further improved by the application of strain B006 (28.6% and 73.5% for treatment combinations LFWB1 and NFWB1, respectively), while other plant hormones under test had no significant difference. Although the ZT contents significantly increased from 5.46 to 7.08 µg g^−1^ at stage T1 to 10.87 to 13.02 µg g^−1^ at stage T2, no significant difference was observed across treatment combinations, as for ABA. Moreover, normal fertigation input combined with application of strain B006 (treatment combination NFWB2) improved the contents of IAA (52.9%), SA (48.6%), and ZT (13.2%) but decreased the ABA contents (30.6%) without significant differences, compared with treatment combination LFWB2.

### 3.4. Rhizosphere Microbial Communities Reshaped by Fertigation and Bacillus Application

**Figure 2:**
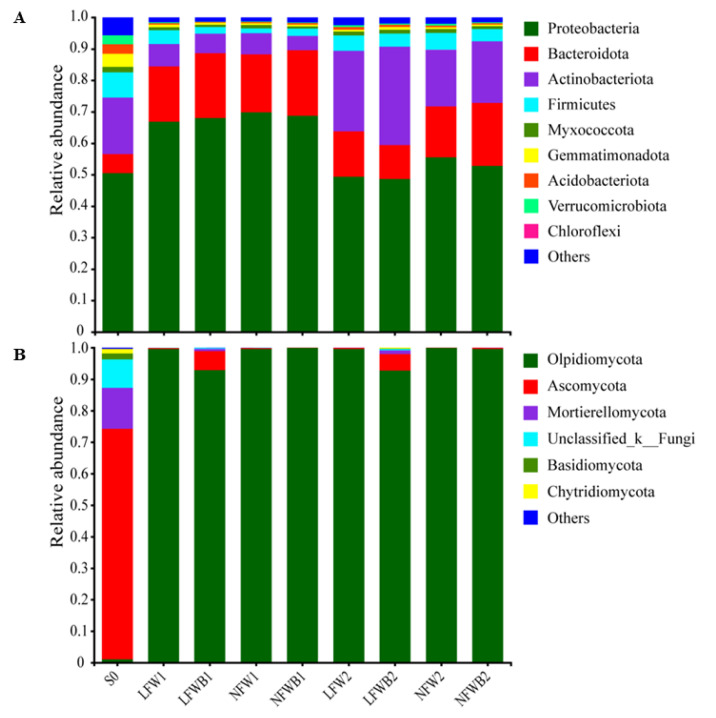
Relative abundance of the predominant bacteria (**A**) and fungi (**B**) in the cabbage rhizosphere soil at phylum level. S0, bulk soil samples collected at time 0; LFW, low fertigation input; NFW, normal fertigation input; B, application with B006; 1 and 2, rhizosphere soil collected at stages T1 and T2, respectively.

The rhizosphere bacterial and fungal communities of cabbage were deciphered by using the high-throughput sequencing platform. A total of 2,230,338 bacterial raw reads and 3,755,380 fungal raw reads were obtained from 36 soil samples and a total of 16,568 bacterial sequences and 57,565 fungal sequences were yielded after evenness analysis (Table S2). After taxonomy assignment with a confidential threshold of 70% and removing ASVs of singletons, mitochondria, and chloroplasts, 4841 distinct bacterial ASVs belonging to 34 phyla, 330 families, and 653 genera and 856 distinct fungal ASVs belonging to 12 phyla, 123 families, and 243 genera were identified. As shown in Figure 2A, Proteobacteria, Bacteroidota, Actinobacteriota, and Firmicutes were the predominant bacteria in all samples at the phylum level, showing relative abundances of 48.62–69.83%, 6.05–20.53%, 4.52–31.31%, and 1.52–7.99%. Throughout cabbage sowing, sprouting, and growing, the bacterial community compositions showed different changing patterns. In comparison with the top ten bacterial phyla in bulk soil, the relative abundances of Proteobacteria and Bacteroidota increased while those of Actinobacteriota and Firmicutes decreased at stage T1. In response to water stress, the relative abundances of Proteobacteria and Bacteroidota decreased, while those of Actinobacteriota increased up to 31.31% when combined with the application of B006 in the cabbage rhizosphere. A similar disturbance occurred in the fungal communities with agricultural practices, as the predominant fungal phyla shifted from Ascomycota (73.23%), Mortierellomycota (13.08%), and unclassified_k_fungi in bulk soil to Olpidiomycota (92.72–99.93%) in treated soil (Figure 2B). Ascomycota with relative abundances of 6.03% and 5.23% became the second top fungal phylum in the rhizosphere soil treated with the combination of low fertigation and B006 application at stages T1 and T2, respectively. 

**Figure 3:**
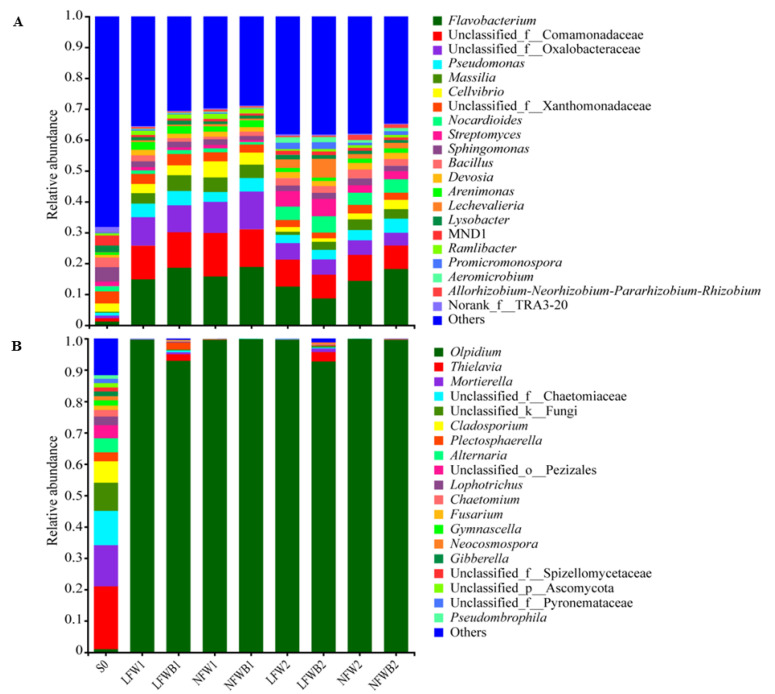
Relative abundance of the predominant bacteria (**A**) and fungi (**B**) in the cabbage rhizosphere soil at genus level. S0, bulk soil samples collected at time 0; LFW, low fertigation input; NFW, normal fertigation input; B, application with B006; 1 and 2, rhizosphere soil collected at stages T1 and T2, respectively.

At the genus level, the bacterial composition and diversity were much more complex in bulk soil, as the total relative abundance of the top 21 genera was less than 35.0% (Figure 3A). In treated rhizosphere soil, *Flavobacterium*, unclassified_f_Comamonadaceae, and unclassified_f_Oxalobacteraceae were predominant at stage T1, with a relative abundance of 25.82% to 43.31% altogether. These genera were still predominant at stage T2, but the sum of their abundances decreased to 21.34–29.92%. B006 application enriched some specific genera in soil at stage T2, such as *Lechevalieria* (6.07%), *Nocardioides* (5.27%), *Streptomyces* (5.63%), and *Massilia* (2.58%) in LFWB2 samples and *Pseudomonas* (4.57%), *Cellvibrio* (2.91%), and *Nocardioides* (4.36%) in NFWB2 samples. The shifts in fungal communities in bulk and treated soil were contrary to those of bacteria. In comparison to the complex structure of the fungal community in bulk soil which was dominated by *Thielavia* (20.09%), *Mortierella* (13.07%), and unclassified_f_Chaetomiaceae (10.95%), *Olpidium* was the extraordinarily predominant genus with relative abundances of 92.72–99.93% in treated soil (Figure 3B). Low fertigation input combined with B006 application enriched *Thielavia*, *Mortierella*, *Plectosphaerella*, and unclassified_f_ Chaetomiaceae in the cabbage rhizosphere at stages T1 and T2.

**Figure 4:**
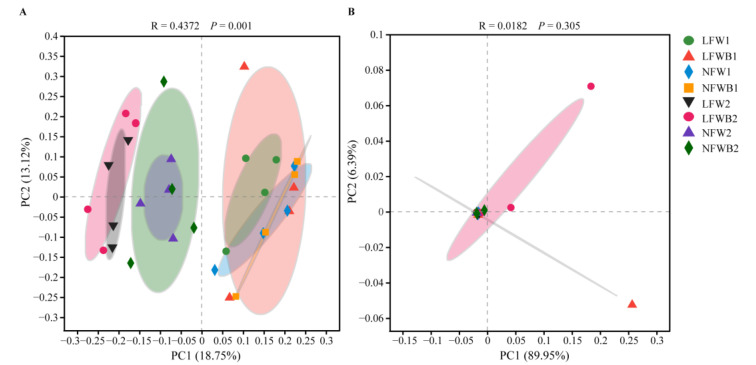
PCoA of the communities of bacteria (**A**) and fungi (**B**) in cabbage rhizosphere soil. LFW, low fertigation input; NFW, normal fertigation input; B, application with B006; 1 and 2, rhizosphere soil collected at stages T1 and T2, respectively; The different color circles in the figure indicate different sample group.

PCoA separated the bacterial and fungal communities into four and two clusters, respectively (Figure S2). The source of soil samples could explain 22.49% (*p* = 0.001) and 74.72% (*p* = 0.003) of the variation of the bacterial and fungal community, respectively, which indicated that the soil source was the main contributor. Further PCoAs on the rhizosphere bacterial communities showed that the four bacterial clusters corresponded to the communities in the rhizosphere soil collected at stage T1 and at stage T2 after treating with low or normal fertigation inputs (Figure 4A and Figure S2A). Further PERMANOVA confirmed that water stress was the main driver of the rhizosphere bacterial community at stage T2 (Table S3). In contrast, fungal communities only showed a distinction between bulk soil and treated soil (Figure 4B and Figure S2B).

**Figure 5:**
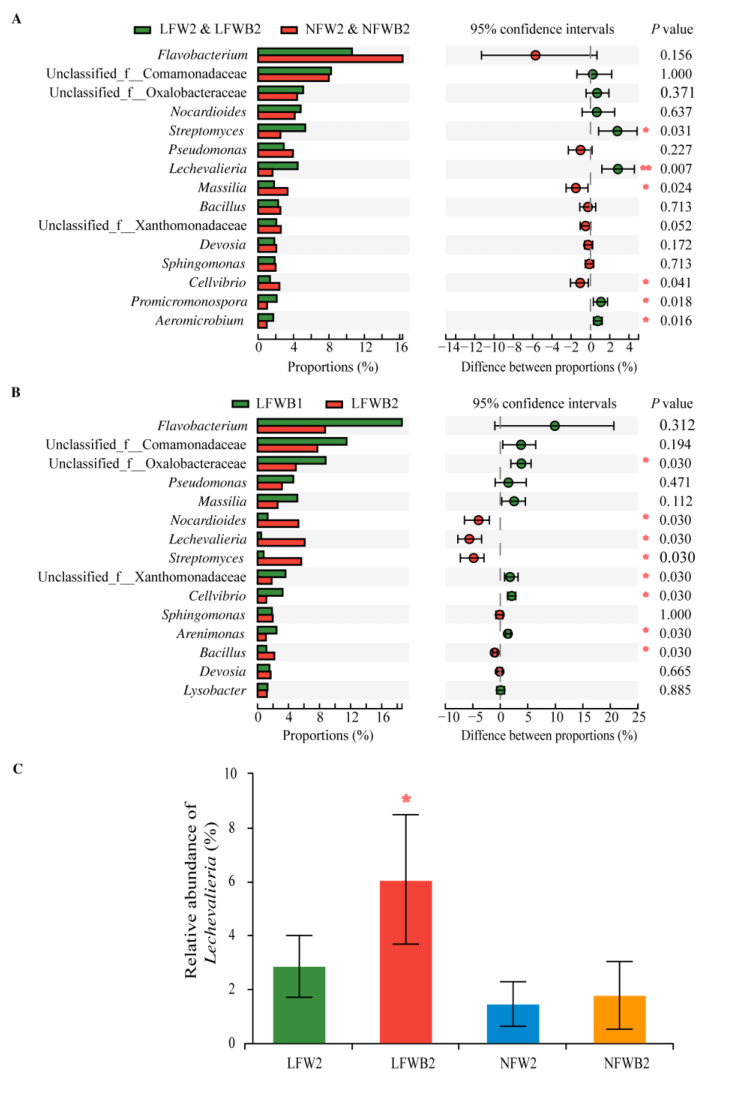
Differential bacteria generated from the pair-wise comparison of groups LFW2 and LFWB2 vs. NFW2 and NFWB2 (**A**) and LFWB2 vs. LFWB1 (**B**). The “*” and “**” on the right side of the columns in (**A**,**B**) or above the column in (**C**) indicate significant and very significant differences at 0.05 and 0.01 levels, respectively. LFW, low fertigation input; NFW, normal fertigation input; B, application with B006; 1 and 2, rhizosphere soil collected at stages T1 and T2, respectively.

Besides community analysis, pair-wise comparisons corresponding to fertigation and microbial application were also conducted to reveal the variations of bacteria (Figure 5). Of the 15 differential genera, no bacterial genera were differential among the pair groups LFW and NFW vs. LFWB and NFWB at stages T1 and T2 (Figure S3), but low fertigation input resulted in the significant enrichment of *Streptomyces*, *Lechevalieria*, *Promicromonospora*, and *Aeromicrobium* (Figure 5A). When combined with B006 application, the relative abundances of *Nocardioides*, *Lechevalieria*, *Streptomyces*, and *Bacillus* were increased significantly at stage T2 (Figure 5B), and those of *Lechevalieria* in LFWB2 samples were much higher than that in other samples (6.07% vs. 1.47–2.87%) (Figure 5C). Taxonomy identification confined 9686 sequences to the 16 ASVs of *Lechevalieria*, and 41.9% of them (4023 sequences) were retrieved from LFWB2 samples. Of the *Lechevalieria* ASVs in LFWB2 samples, ASV1 (58.45%) and ASV14 (34.28%) were predominant, and eight ASVs (ASV108, ASV3710, ASV315, ASV3701, ASV4118, ASV364, ASV284, and ASV4173) were unique (Table S4).

### 3.5. Correlation of the Rhizosphere Bacteria to Plant Growth, Fertigation Levels, and Hormones

**Figure 6:**
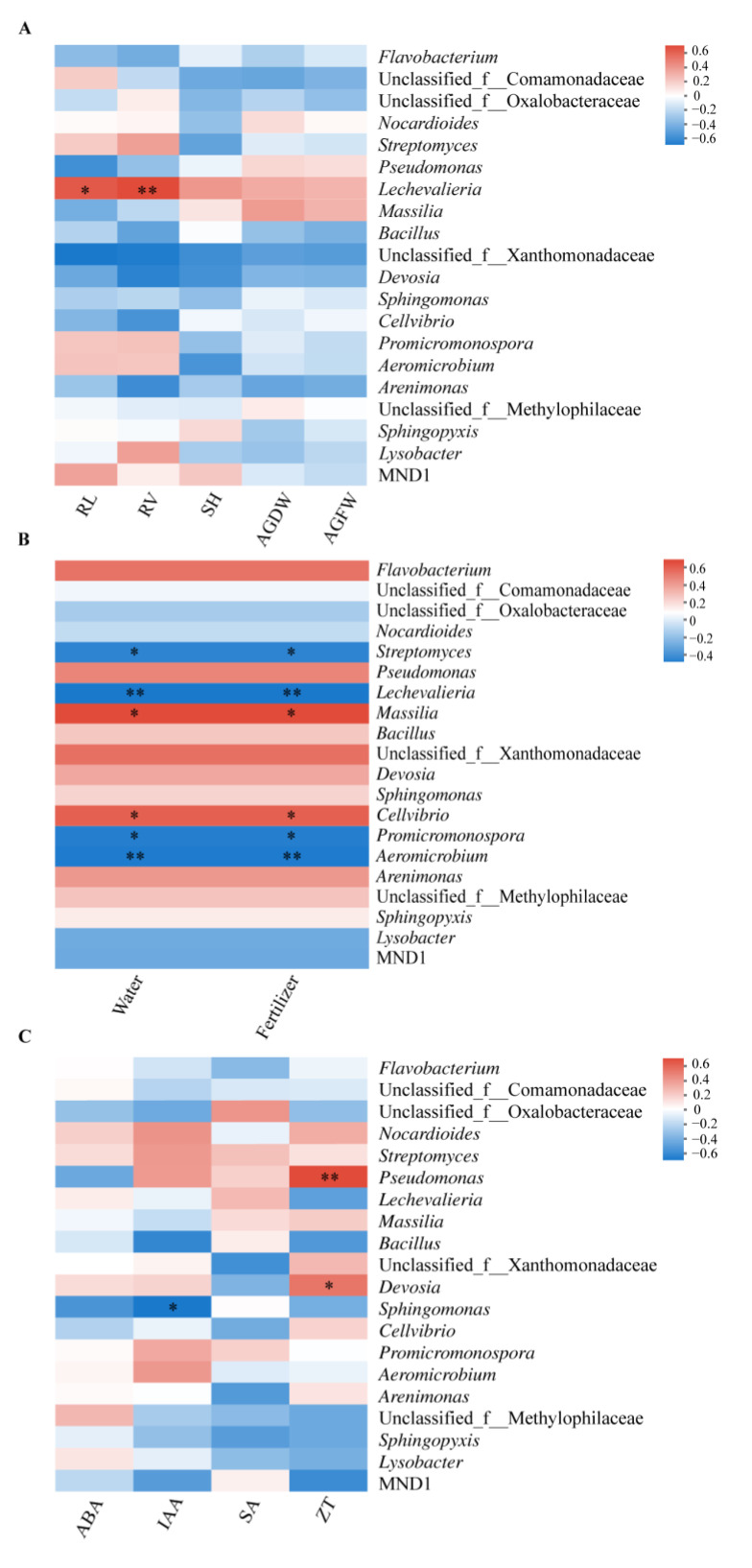
Heatmap analyses to visualize the correlation of rhizosphere bacteria with cabbage growth (**A**), agricultural practices (**B**), and root hormone production (**C**). RL, root length; RV, root vitality; SH, seedling height; AGDW, the above-ground dry weight; AGFW, the above-ground fresh weight; ABA, abscisic acid; IAA, indole-3-acetic acid; SA, salicylic acid; ZT, zeatin; “*” and “**” in the figure indicate significant and very significant difference at 0.05 and 0.01 levels, respectively.

Of the 20 top rhizosphere bacterial genera, only *Lechevalieria* was found to be positively correlated with the root length (*p* < 0.05) and root vitality (*p* < 0.01) significantly (Figure 6A). Although water and fertilizer inputs showed similar effects on the rhizosphere bacteria, the responses of different bacteria varied a lot (Figure 6B). *Streptomyces*, *Lechevalieria*, *Promicromonospora*, and *Aeromicrobium* were negatively correlated to the normal fertigation input (water potential of −30 kPa and fertilizer of 165.25 kg hm^−2^), while *Massilia* and *Cellvibrio* were positively correlated. As to the correlation of rhizosphere bacteria to four tested plant hormones, it was found that *Nocardioides*, *Streptomyces*, *Pseudomonas*, *Promicromonospora*, and *Aeromicrobium* were positively related to IAA and unclassified_f_Comamonadaceae, *Streptomyces*, and *Lechevalieria* were positively related to SA, although the relationships were insignificant, while positive correlation was only observed between *Pseudomonas* and ZT (*p* < 0.01) and *Devosia* and ZT (*p* < 0.05) (Figure 6C).

## 4. Discussion

### 4.1. Healthy Cabbage Growth Promoted by Improved Bacillus Application

Soft rot disease often occurs in Chinese cabbage fields, rapidly spreads due to overirrigation or excess nitrogen input, and is hard to control [1,23,36]. In this study, low fertigation input was adopted to grow Chinese cabbage in fields. In comparison with normal fertigation conditions (29.75 Kg hm^−2^, 8.26 Kg hm^−2^, 21.48 Kg hm^−2^, and 625.8 m^3^ hm^−2^ for nitrogen, phosphorous, potassium, and water, respectively), the total amounts of water and fertilizer were decreased by 22.7% to 62.0%. However, low fertigation input had no significant effect on the growth performance and root development of cabbage seedlings (LFW2 vs. NFW2; Figure 1). The reason might be the short duration of water stress (10 days) before statistically significant phenotypic changes were observed. When cabbage was infested with the soft rot pathogen, low fertigation input restrained the occurrence and development of soft rot disease and enhanced the yield (LFW vs. NFW; Table 2).

*B. velezensis* B006 is a biocontrol bacterium with antagonistic activity against fungal and bacterial pathogens [39,40]. Its integration with low fertigation input not only promoted plant growth (LFWB2 vs. other treatments; Figure 1) but also enhanced the control efficacy of soft rot disease (80.9% of LFWB vs. 8.5% of NFWB and 56.7% of LFW) and cabbage yield (4.3 × 10^4^ Kg hm^−2^ of LFWB vs. 3.5–3.8 × 10^4^ Kg hm^−2^ of NFW and NFWB). The results suggest that B006 is a plant growth-promoting rhizobacterium (PGPR) that functions through increasing nutrient bioavailability in soil and plant productivity and minimizing the fertilizer input to a certain extent [56,57,58]. Therefore, the integrated application of low fertigation input and B006 represents a cost-effective and control-efficient practice for vegetable production and disease control.

### 4.2. Specific Assembly of Bacterial Genera Shaped by Improved Bacillus Application

The rhizosphere is the plant niche harboring diverse soil microbes to respond to various biotic and abiotic stresses, and the rhizosphere microbiota is shaped by abiotic factors such as water content, fertilizer, tillage, as well as biotic factors like PGPR introduction and pathogen invasion. Drought stress can promote the enrichment of *Streptomyces* in the rhizosphere, alleviate the negative effect of drought, and improve plant growth and yield [27,29,59], while low levels of nitrogen and phosphorus can promote the enrichment of *Acidobacteria*, *Chloroflexia*, and *Bacillus* [60]. In comparison with the dynamics of bacterial communities, fungal communities are more stable and resistant to environmental stresses [61]. In this study, the diversity and composition of the cabbage rhizosphere microbial communities were analyzed before and after treating with low or normal fertigation inputs and with or without B006 application. It was found that low fertigation input did enrich *Streptomyces* in the cabbage rhizosphere in response to water stress (Figure 3A and Figure 5A) as reported previously but had no similar effects on the abundances of *Acidobacteria*, *Chloroflexia*, and *Bacillus* upon nutrient starvation. Instead, *Lechevalieria*, *Promicromonospora* and *Aeromicrobium* were enriched under low-fertigation conditions. *Lechevalieria* was previously isolated from a wheat rhizosphere [62] and could produce various active metabolites [63]. *Promicromonospora*, sporangium-forming actinomycetes, have been isolated from various niches (soil, gut, sea water, etc.) and demonstrate PGPR activities. For example, *Promicromonospora* sp. SE188 producing gibberellin can improve the growth of *Solanum lycopersicum* and influence the production of endogenous plant hormones [64]. *Aeromicrobium*, a genus of actinomycetes, also has the potential of control plant disease by producing antimicrobial molecules [65]. A *Streptomyces* strain, 26B, isolated from water-stressed soil has been confirmed to have the capability of promoting cabbage seedling growth and suppressing soft rot disease by antagonistic metabolites under drought [66]. Therefore, we infer that enriched *Streptomyces*, *Lechevalieria*, *Promicromonospora* and *Aeromicrobium* in the cabbage rhizosphere may function together to play multiple beneficial roles through producing active metabolites or antimicrobial molecules.

In addition to the abiotic factors like water stress and nutrient starvation discussed above, biotic factors like introduction of PGPR also shaped the rhizosphere bacterial communities. Application of bio-organic fertilizer containing *Bacillus* and *Trichoderma* is efficient to suppress Vanilla fusarium wilt through the recruitment of *Lysobacter* in the soil [67]. Co-inoculation of several *Pseudomonas* strains directly affected the composition, diversity, and function of the native soil bacterial community and promoted plant growth through re-construction of the bacterial community [68]. Sun et al. [69] found that inoculation of *Bacillus velezensis* could stimulate the resident rhizosphere bacterium *Pseudomonas stutzeri* and this two-species consortium to promote plant growth and help plants alleviate salt stress. Co-occurrence of soil microbes also has been reported to have synergistic effects on wheat growth and microbe-mediated nitrogen nutrient cycling (release, loss, absorption, etc.); of them, *Nocardioides* is closely related to denitrification reactions [70]. In our previous study, application of *B. velezensis* B006 under low fertigation could promote eggplant to absorb nutrients from soil and finally facilitate eggplant growth [37]. In this study, we found that application of B006 promoted cabbage growth and enriched the abundances of *Lechevalieria*, *Nocardioides*, and *Streptomyces* in the cabbage rhizosphere at stage T2 instead of stage T1 (LFWB2 vs. LFWB1; Figure 5B) and their co-occurrence might improve cabbage growth through denitrification to reduce the negative effects of NO_3_^−^ accumulation [70] or through promoting the absorption of available K and P from soil [37]. Overall, these results suggested that the integrated application of optimal fertigation and beneficial agents is efficient to reshape the rhizosphere bacterial communities against biotic and abiotic stresses and promote the healthy and sustainable growth of plants.

### 4.3. Enrichment of Specific Bacterial Genera and Hormone Production Are Positively Related to the Improved Bacillus Application and Healthy Cabbage Growth

The plant microbiome is one of the key determinants of plant health and productivity [71]. Its versatile effects are roughly divided into two fractions [72]: one is to improve plant adaptability and fitness in response to environmental changes, especially abiotic (e.g., drought) or biotic (e.g., herbivores) stress, and the other is to support plant growth through altering root system architecture, such as stimulating lateral root growth and root hair formation, which further improves water and nutrient accessibility [73]. In this study, application of a *Bacillus* agent changed the rhizosphere soil microbiota to promote cabbage growth and defense and to adapt to water stress (Figure 1 and Figure 4). Of the enriched rhizosphere bacteria, *Lechevalieria* was the unique genus that was significantly positively related to the plant growth (root vitality and root length) and negatively related to high fertigation input (Figure 6), suggesting that this genus might play important roles in alleviating water stress and promoting cabbage growth. It provides ideas to improve plant performance and defense by engineering the soil and plant microbiome together.

Plant hormones are considered as the most important chemical messengers to communicate between plants and the plant microbiome. They regulate the microbial diversity in the endosphere and different root compartments to improve plant defense and development or in the rhizosphere to exert direct or indirect activities [74]. Of the plant hormones, abscisic acid, indole-3-acetic acid, salicylic acid, and zeatin (a kind of cytokinin) have been reported to promote plant resistance against drought, low temperature, etc. [75]. Abscisic acid has activities to assist plants to absorb soil water and to promote root development to defend against drought [76,77,78] and is well known to induce plant systemic resistance. Indole-3-acetic acid is also important for root development through interacting with other hormones [79], and its content is increased in maize roots under drought stress [80]. Cytokinin can regulate root gravitropism, root elongation, and lateral roots development [81,82,83]. In this study, the contents of salicylic acid were higher at stage T1, zeatin contents were higher at stage T2 (Table 2), and the indole-3-acetic acid contents were higher in *Bacillus*-treated samples. This indicates that the production of different hormones in cabbage roots is closely related to the growing stage and treatments. Moreover, it was found that the salicylic acid content was further increased by the application of *Bacillus* in all samples except when B006 was added to LFW. A previous study has shown that PGPBs can activate induced systemic resistance via salicylic acid- or jasmonic acid (and ethylene)-dependent signaling pathways to improve plant resistance against drought or salinity [84]. The distinction might be ascribed to the level of drought stress inducing different hormones and exudates in cabbage roots that reshape the bacterial communities [85]. Correlation analysis showed that the contents of indole-3-acetic acid and salicylic acid were related to the abundance of some actinomycetes in the rhizosphere under low-fertigation conditions, but the correlation was insignificant (Figure 6C). This means that a simple correlation of different factors is far from the actual underlying interactions of plant, plant microbiome, and environment [65]. Further studies on the co-occurrence of plant microbiome indicators and key plant traits will be conducted to decipher the mechanism of soil–plant–microbe interactions, regulate the plant beneficial microbiome, and promote plant growth for the purpose of sustainable and cost-effective production.

## 5. Conclusions

Combined application of *Bacillus* with low fertigation input promoted cabbage growth and increased root vitality, which is positively related to the enrichment of *Lechevalieria* in the cabbage rhizosphere and the production of indole-3-acetic acid and salicylic acid in cabbage roots. Moreover, decreased input of water and fertilizer is efficient to reduce the occurrence of soft rot disease and increase the yield, thus representing a practical and cost-effective soil tillage strategy. This study gives new and practical evidence on the improvement of *Bacillus* application in fields to promote the healthy growth and productivity of cabbage.

## Figures and Tables

**Table 1:** Criteria for the classification of Chinese cabbage soft rot disease in field.

Disease Level	Symptoms
0	No disease
1	Soft rot scab appeared on the lower 1–2 leaves
2	Soft rot scab appeared on the lower 3 leaves
3	Plants wilted, large areas of soft rot scab on outside leaves, the plant growth was seriously retarded
4	Stem soft rot, whole plant died

**Table 2:** Effect of combined *B. velezensis* B006 and low fertigation input on the occurrence of soft rot disease and yield of Chinese cabbage at stage T3.

Treatment ^a^	Disease Index ^b^	Control Efficacy (%)	Seedling Survival Rate (%)	Yield (×10^4^ Kg hm^−2^)
LFW	6.1 ± 2.1 b	56.7	86.6 ± 3.0 a	4.1 ± 0.3 ab
LFWB	2.7 ± 1.1 b	80.9	89.2 ± 4.4 a	4.3 ± 0.3 a
NFW	14.1 ± 3.1 a	–	67.1 ± 5.7 b	3.5 ± 0.4 c
NFWB	12.9 ± 2.2 a	8.5	71.6 ± 7.4 b	3.8 ± 0.2 bc

^a^ LFW, low fertigation input; NFW, normal fertigation input; B, application with B006. ^b^ Different letters of the same column indicate significant difference at 0.05 level.

**Table 3:** Contents of endogenous hormones in cabbage roots determined by using HPLC ^a^.

Samples ^b^	ABA (µg g^−1^)	IAA (µg g^−1^)	SA (µg g^−1^)	ZT (µg g^−1^)
LFW1	0.60 ± 0.32 ab	– ^c^	14.33 ± 2.61 b	5.46 ± 0.80 b
LFWB1	1.00 ± 0.15 a	0.42 ± 0.25 bc	18.43 ± 0.95 a	7.08 ± 1.85 b
NFW1	0.88 ± 0.20 ab	–	9.83 ± 2.70 bc	6.32 ± 0.69 b
NFWB1	0.84 ± 0.27 ab	0.34 ± 0.07 c	17.05 ± 2.45 a	6.19 ± 1.01 b
LFW2	0.65 ± 0.34 ab	0.30 ± 0.01 c	6.04 ± 0.67 d	10.87 ± 1.40 a
LFWB2	0.72 ± 0.34 ab	0.70 ± 0.28 ab	5.08 ± 1.15 de	11.50 ± 2.19 a
NFW2	0.63 ± 0.19 ab	–	3.31 ± 1.00 e	11.42 ± 0.89 a
NFWB2	0.50 ± 0.24 b	1.07 ± 0.48 a	7.55 ± 0.50 cd	13.02 ± 1.77 a

^a^ ABA, abscisic acid; IAA, indole-3-acetic acid; SA, salicylic acid; ZT, zeatin. ^b^ LFW, low fertigation input; NFW, normal fertigation input; B, application with B006; 1 and 2, collected at stages T1 and T2, respectively; ^c^ Not detected; Different letters following the data indicate significant difference at 0.05 level.

## Data Availability

All data were published in this article.

## Supplementary Materials

The following supporting information can be downloaded at: https://www.mdpi.com/article/10.3390/biology12081130/s1, Figure S1: Chromatograms and standard curves of plant hormones detected by HPLC at 254 nm. Figure S2: PCoA of the communities of bacteria (A) and fungi (B) in the bulk soil and cabbage rhizosphere soil. Figure S3: Differential bacterial genera generated from the pair-group comparison of groups LFWB1 and NFWB1 vs. LFW1 and NFW1 (A) and LFWB2 and NFWB2 vs. LFW2 and NFW2 (B). Table S1: Experimental design of different fertigation treatments combined with *Bacillus* application. Table S2: Amounts of bacterial and fungal reads and ASVs of the microbial communities in each soil sample. Table S3: PERMANOVA on the rhizosphere bacterial communities of cabbage collected at stage T2. Table S4: *Lechevalieria* ASVs identified in the cabbage rhizosphere of different treatment combinations.
