# Supplementary material for: Combination of Bacillus and Low Fertigation Input Promoted the Growth and Productivity of Chinese Cabbage and Enriched Beneficial Rhizosphere Bacteria Lechevalieria
Source: Biology (Basel). 2023 Aug 14;12(8):1130. doi: 10.3390/biology12081130 (PMC10452305; doi:10.3390/biology12081130)
Supplement: Supplementary file 1 [file biology-12-01130-s001.zip › biology-2491680-supplementary.pdf]

Supplementary materials to the Article

# Combination of *Bacillus* and Low Fertigation Input Promoted the Growth and Productivity of Chinese Cabbage and Enriched Beneficial Rhizosphere Bacteria *Lechevalieria*

Shi-Chang Zhang [1,2], Yu-Lu Zhang [1], Xiao-Jing Guo [1], Ming Luo [2], Shi-Dong Li [1] and Rong-Jun Guo [2,*]

[1] State Key Laboratory for Biology of Plant Diseases and Insect Pests, Institute of Plant Protection, Chinese Academy of Agricultural Sciences, Beijing 100193, P. R. China;

[2] College of Agriculture, Xinjiang Agricultural University, Wulumuqi 830052, P. R. China;

[*] Correspondence: guorongjun@caas.cn

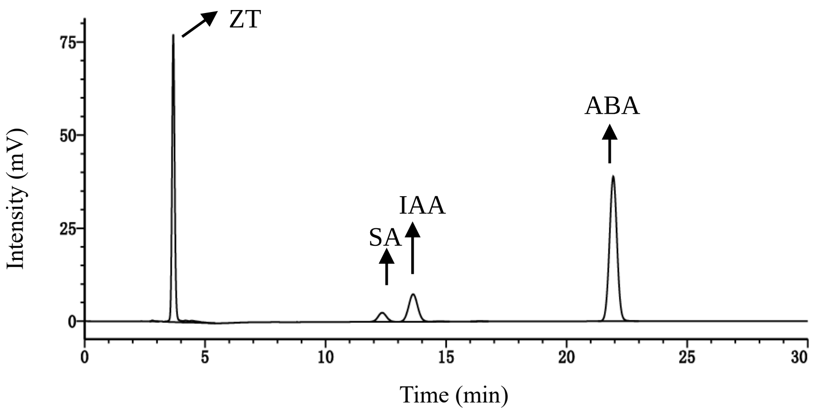


| Hormone | Concentration (μg mL$^{-1}$) | Linear equation | Correlation coefficient | Detection limit (ng mL$^{-1}$) |
|---|---|---|---|---|
| ABA | 0.05–1.0 | y = 49115x–288.82 | 1.0000 | 20.0 |
| IAA | 0.2–10.0 | y = 7325.1x–139.57 | 1.0000 | 100.0 |
| SA | 1.0–40.0 | y = 2596.2x–1230.1 | 0.9991 | 400.0 |
| ZT | 1.0–40.0 | y = 28662x–23967 | 0.9983 | 10.0 |

**Figure S1** Chromatograms and standard curves of plant hormones detected by HPLC at 254 nm. ABA, abscisic acid; IAA, indole-3-acetic acid; SA, salicylic acid, and ZT, zeatin. Linear equation, x and y represent the concentration and relative peak area of each plant hormone respectively.

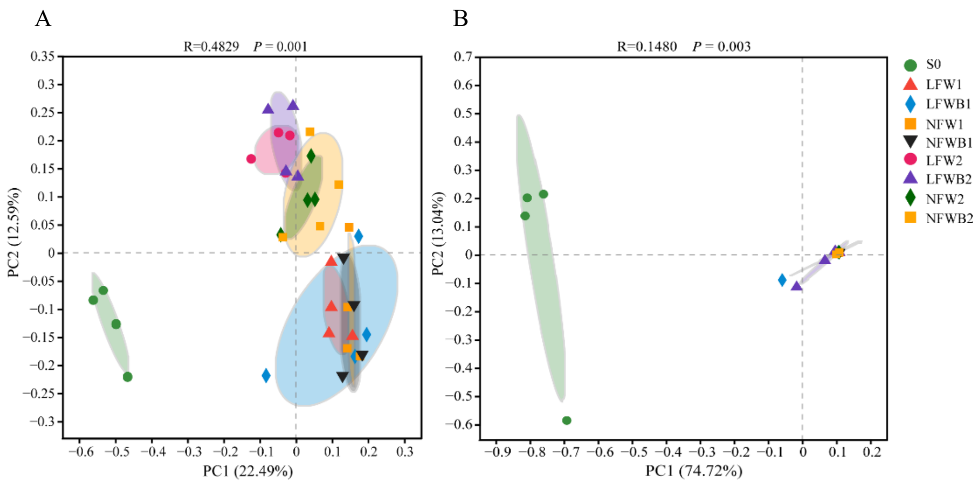


**Figure S2** PCoA analysis of the communities of bacteria (A) and fungi (B) in the bulk soil and cabbage rhizosphere soil.

S0, bulk soil samples collected at time 0; LFW, low input fertigation; NFW, normal input fertigation; B, with B006 application; 1 and 2, rhizosphere soil collected at stages T1 and T2, respectively.

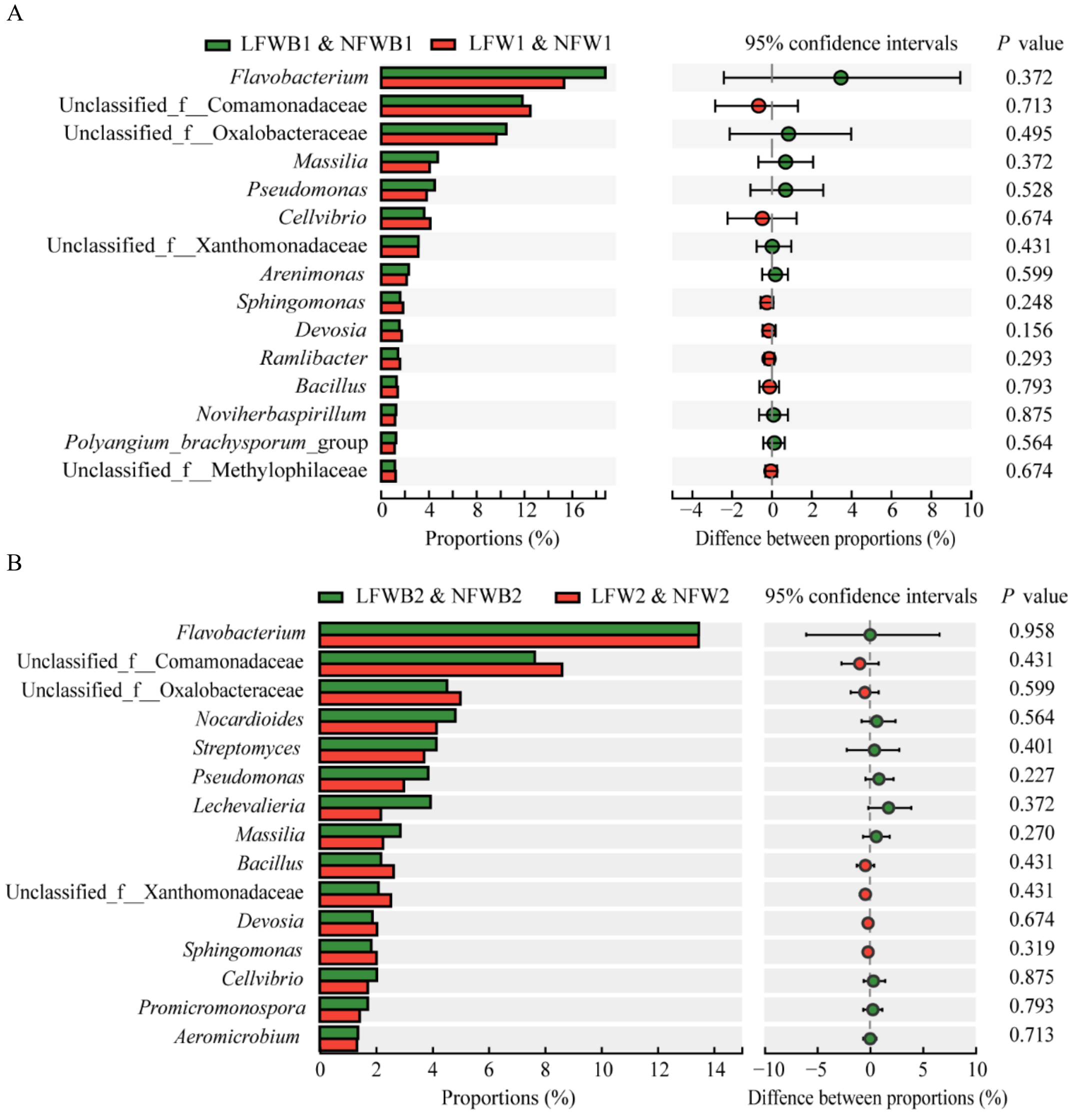


**Figure S3** Differential bacterial genera generated from the pair-group comparison of groups LFWB1 & NFWB1 vs. LFW1 & NFW1 (A) and LFWB2 & NFWB2 vs. LFW2 & NFW2 (B).

LFW, low input fertigation; NFW, normal input fertigation; B, application with B006; 1 and 2, rhizosphere soil collected at stages T1 and T2, respectively.

**Table S1** Experimental design of different fertigation treatments combined with *Bacillus* application.[a]

| Treatment[b] | S0 | T1 | | | T2 | | | T3 | | |
|---|---|---|---|---|---|---|---|---|---|---|
| | | Soil water potential (kPa) | Practical irrigation (m$^3$ hm$^{-2}$) | Amount of water soluble fertilizer (Kg hm$^{-2}$) | Soil water potential (kPa) | Practical irrigation (m$^3$ hm$^{-2}$) | Amount of water soluble fertilizer (Kg hm$^{-2}$) | Soil water potential (kPa) | Practical irrigation (m$^3$ hm$^{-2}$) | Amount of water soluble fertilizer (Kg hm$^{-2}$) |
| LFW<br>LFWB | Before planting | –30 | 199.1 | –[c] | –50 | 26.7 | 62.50 | –45 | 257.8 | – |
| NFW<br>NFWB | | –30 | 199.1 | – | –30 | 53.3 | 165.25 | –25 | 373.3 | – |

[a] S0, bulk soil samples collected at time 0; T1, T2, and T3, rhizosphere soil collected at stages T1,T2, and T3, respectively.

[b] LFW, low fertigation input; NFW, normal fertigation input; B, application with B006.

[c] –, non application.

**Table S2** Amounts of reads and ASVs of the microbial communities in each soil sample.

| Group name [a] | Sample name | Bacteria | | Fungi | |
|---|---|---|---|---|---|
| | | Reads | ASVs | Reads | ASVs |
| S0 | S01 | 54070 | 850 | 67073 | 296 |
| | S02 | 61119 | 579 | 70907 | 369 |
| | S03 | 67832 | 1012 | 67963 | 123 |
| | S04 | 65163 | 798 | 61486 | 246 |
| LFW1 | LFW11 | 50847 | 483 | 75253 | 29 |
| | LFW12 | 73934 | 590 | 80822 | 11 |
| | LFW13 | 59860 | 551 | 123508 | 14 |
| | LFW14 | 54764 | 488 | 127314 | 12 |
| LFWB1 | LFWB11 | 51749 | 477 | 104343 | 97 |
| | LFWB12 | 58491 | 463 | 117311 | 8 |
| | LFWB13 | 40613 | 339 | 106083 | 22 |
| | LFWB14 | 51265 | 387 | 88739 | 8 |
| NFW1 | NFW11 | 50644 | 425 | 106180 | 19 |
| | NFW12 | 46292 | 328 | 109460 | 28 |
| | NFW13 | 63263 | 545 | 118606 | 13 |
| | NFW14 | 47175 | 401 | 102315 | 24 |
| NFWB1 | NFWB11 | 50454 | 453 | 101350 | 10 |
| | NFWB12 | 43308 | 345 | 102499 | 7 |
| | NFWB13 | 73368 | 538 | 108119 | 17 |
| | NFWB14 | 58887 | 439 | 103881 | 18 |
| LFW2 | LFW21 | 62799 | 511 | 119588 | 8 |
| | LFW22 | 61566 | 520 | 102902 | 23 |
| | LFW23 | 73168 | 806 | 91147 | 26 |
| | LFW24 | 67553 | 629 | 90658 | 19 |
| LFWB2 | LFWB21 | 66485 | 797 | 119972 | 218 |
| | LFWB22 | 63583 | 532 | 126924 | 132 |
| | LFWB23 | 69762 | 771 | 98820 | 41 |
| | LFWB24 | 73745 | 716 | 101622 | 9 |
| NFW2 | NFW21 | 68692 | 642 | 104026 | 7 |
| | NFW22 | 70827 | 718 | 90111 | 3 |
| | NFW23 | 73539 | 722 | 140323 | 10 |
| | NFW24 | 71075 | 661 | 159750 | 14 |
| NFWB2 | NFWB21 | 70630 | 523 | 122690 | 5 |
| | NFWB22 | 71494 | 697 | 111405 | 59 |
| | NFWB23 | 70307 | 565 | 120908 | 8 |
| | NFWB24 | 72015 | 664 | 111322 | 3 |
| **Total** | | **2230338** | **3755380** | **20965** | **1956** |

[a] S0, bulk soil samples collected at time 0; LFW, low input fertigation; NFW, normal input fertigation; B, application with B006; 1 and 2, rhizosphere soil collected at stages T1 and T2, respectively.

**Table S3** PERMANOVA analysis on the rhizosphere bacterial communities of cabbage collected at stage T2

| Name[a] | Df | Sums of Sqs | Mean Sqs | F. Models | $R^2$ | Pr (>F) |
|---|---|---|---|---|---|---|
| NB vs. B | 1 | 0.100789 | 0.100789 | 0.79477 | 0.049184 | 0.781 |
| LFW vs. NFW | 1 | 0.299825 | 0.299825 | 2.364264 | 0.146312 | 0.001 |
| Residuals | 13 | 1.648602 | 0.126816 | - | 0.804504 | - |
| Total | 15 | 2.049217 | - | - | 1 | - |

[a] LFW, low fertigation input; NFW, normal fertigation input; NB, no B006 application; B, application with B006.

**Table S4** *Lechevalieria* ASVs identified in the cabbage rhizosphere of different treatment combinations [a].

| ASV | No. of sequences | Proportion（%） | T1 | | | | T2 | | | |
|---|---|---|---|---|---|---|---|---|---|---|
| | | | LFW1 | LFWB1 | NFW1 | NFWB1 | LFW2 | LFWB2 | NFW2 | NFWB2 |
| ASV1 | 5621 | 58.45 | 181 | 155 | 331 | 263 | 917 | 2298 | 584 | 892 |
| ASV14 | 3296 | 34.28 | 166 | 141 | 141 | 112 | 910 | 1269 | 266 | 291 |
| ASV13 | 355 | 3.69 | 0 | 0 | 29 | 0 | 0 | 204 | 122 | 0 |
| ASV268 | 101 | 1.05 | 0 | 0 | 0 | 0 | 41 | 60 | 0 | 0 |
| ASV108 | 75 | 0.78 | 0 | 0 | 0 | 0 | 0 | 75 | 0 | 0 |
| ASV2331 | 48 | 0.50 | 0 | 13 | 0 | 0 | 24 | 11 | 0 | 0 |
| ASV3710 | 26 | 0.27 | 0 | 0 | 0 | 0 | 0 | 26 | 0 | 0 |
| ASV315 | 19 | 0.20 | 0 | 0 | 0 | 0 | 0 | 19 | 0 | 0 |
| ASV3701 | 18 | 0.19 | 0 | 0 | 0 | 0 | 0 | 18 | 0 | 0 |
| ASV4118 | 16 | 0.17 | 0 | 0 | 0 | 0 | 0 | 16 | 0 | 0 |
| ASV364 | 14 | 0.15 | 0 | 0 | 0 | 0 | 0 | 14 | 0 | 0 |
| ASV284 | 13 | 0.14 | 0 | 0 | 0 | 0 | 0 | 13 | 0 | 0 |
| ASV4722 | 8 | 0.08 | 0 | 0 | 0 | 0 | 8 | 0 | 0 | 0 |
| ASV4173 | 3 | 0.03 | 0 | 0 | 0 | 0 | 0 | 3 | 0 | 0 |
| ASV2049 | 3 | 0.03 | 0 | 0 | 0 | 0 | 0 | 0 | 0 | 3 |

[a] LFW, low fertigation input; NFW, normal fertigation input; B, application with B006; 1 and 2, rhizosphere soil collected at stages T1 and T2, respectively.
